# How gambling harms experienced by Pacific people in New Zealand amplify when they are culture-related

**DOI:** 10.1186/s40405-017-0026-3

**Published:** 2017-08-18

**Authors:** Komathi Kolandai-Matchett, Erika Langham, Maria Bellringer, Pesio Ah-Honi Siitia

**Affiliations:** 1 Gambling and Addictions Research Centre, Faculty of Health and Environmental Sciences, Auckland University of Technology, Private Bag 92006, North Campus, 90 Akoranga Drive, Auckland, 0627 New Zealand; 2 Centre for Indigenous Health Equity Research, School of Health, Medical and Applied Sciences, CQUniversity Australia, Level 2 Cairns Square, Corner Abbott and Shields Streets, Cairns, QLD 4870 Australia; 3 Problem Gambling Foundation of New Zealand, 128 Khyber Pass Rd, Grafton, Auckland, 1150 New Zealand

**Keywords:** Gambling harms, Pacific cultures, Culture-gambling intersections

## Abstract

Pacific people in New Zealand are a minority ethnic population identified in national prevalence studies as having the highest risk of developing gambling problems. As earlier studies identified some links between culture and gambling for this population, our study aimed to deepen understanding of these links and their role in explaining the disproportionate gambling harms experienced by Pacific people. To achieve this aim we employed intersectionality as a theoretical framework to explore the culture-gambling intersection for this population group. We analysed data from a subset of focus groups conducted for a broad study of gambling harms in New Zealand. The subset was selected based on the presence of individuals knowledgeable about Pacific people’s gambling behaviours, including staff from Pacific problem gambling treatment services who provided examples from a cultural perspective. We identified themes at a latent level via an interpretive process to identify underlying cultural contexts of gambling harms. Findings indicated that whilst harms experienced by Pacific people were similar to those identified amongst the general population, the cultural contexts in which some harms manifested were complex. This paper contributes to the existing knowledge base about gambling harms for Pacific people in relation to six culture-gambling intersecting themes that emerged from the data: collectivism, gift-giving, gambling-based fundraising, patriarchy, beliefs about blessings, and sports celebrities. Findings are discussed in relation to the current knowledge of gambling and conceptualisations of gambling harm within Pacific communities. Implications for culturally appropriate harm minimisation strategies and prevention interventions for this population are suggested.

## Background

New Zealand’s census data indicate a steady growth in the Pacific population (Statistics New Zealand and Ministry of Pacific Island Affairs 2010); the 295,941 individuals in 2013 (7.4% of the population) (Statistics New Zealand 2013) is projected to reach 480,000 by 2026 (Bascand 2010). National surveys have shown that Pacific people^1^ have lower gambling participation relative to the general population; however, those who do gamble tend to have a markedly higher risk of developing gambling problems (Abbott et al. 2014; Abbott 2001; Abbott and Volberg 2000; Ministry of Health 2006, 2009, 2012). In a nationally representative study conducted in 2012 (N = 6251), 8% of Pacific people were classified as moderate-risk or problem gamblers (Abbott et al. 2014). Pacific people (32%) also reported adverse financial consequences from someone else’s gambling more often than other ethnicities (Abbott et al. 2014). A similar finding was noted in a nationally representative sample of New Zealand secondary school students (Rossen et al. 2016).

The consistency in findings from different studies over the last two decades confirms the persistence of high risk of gambling-related harm experienced by Pacific people. This persistence combined with a gradual increase in the size of New Zealand’s Pacific population raises concerns about a potential increase in burden of gambling harms experienced by this population and New Zealand society as a whole. Although gambling-related help seeking among Pacific people remains low (Ministry of Health 2008a), there have been ongoing increases in help sought via the Gambling Helpline (Ministry of Health 2006, 2008b) which could be linked to increasing public health activities on raising the awareness of gambling harms, and the formation of several Pacific-specific gambling treatment services, over the past decade. This supports the value of enhancing health sector understanding of gambling harms among Pacific people and the necessity for culturally appropriate harm minimisation strategies and prevention interventions.

Given the effect cultural beliefs and values can have on gambling-related perceptions and gambling behaviour (see Ji et al. 2015; Kim 2011; Ohtsuka 2013; Raylu and Oei 2004; Sobrun-Maharaj et al. 2012; Subramaniam et al. 2015; Tse et al. 2012; Urale et al. 2015), an accurate understanding of gambling harm among different ethnic communities requires an understanding of the cultural contexts in which gambling behaviour occurs and manifests harm. Some studies have identified that gambling harms experienced by Pacific people include negative effects on health, finances, employment and education; disruptions to household and extended family relationships; unfulfilled childcare; reduced community contributions; and unmet responsibilities being passed on to family members (Bellringer et al. 2013; Guttenbeil-Po’uhila et al. 2004; Lin et al. 2011; Perese and Faleafa 2000). Lin et al. (2011) highlighted how Pacific people, alongside New Zealand’s indigenous Māori, differed from other ethnicities. Within a sample of 1031 Pacific people, the authors found significant associations between gambling participation and poorer self-ratings on quality of life indicators (physical health, mental wellbeing, financial situation and, overall life satisfaction). However, there is a dearth of empirical research exploring the underlying reasons for the elevated gambling-related risk among Pacific people and only a few studies have investigated how gambling participation is linked with Pacific cultural beliefs and practices (Bellringer et al. 2006, 2013; Guttenbeil-Po’uhila et al. 2004; Perese and Faleafa 2000; Tse et al. 2012). A longitudinal study of Pacific mothers in New Zealand found that those who engaged in cultural gift-giving practices were more likely to gamble and have a higher weekly gambling expenditure—behaviours likely to lead to a higher risk of developing gambling problems (Bellringer et al. 2006). At a later data collection point of this longitudinal study, the link between gift-giving and higher gambling participation was again evidenced among Pacific mothers (Perese et al. 2011).

Raylu and Oei (2004) noted that the lack in empirical data on possible links between cultural variables and gambling behaviours prevents a systematic data-driven framework, and that any hypothesised links need to be first explored before they can be embedded within a theoretical framework. Nevertheless, past studies (Bellringer et al. 2006, 2013; Guttenbeil-Po’uhila et al. 2004; Perese and Faleafa 2000; Tse et al. 2012) have hinted at the existence of such links among Pacific communities. Our exploratory study is a preliminary step towards deepening understanding of these culture-gambling links and their role in explaining the disproportionate gambling harms experienced by Pacific people. Employing *intersectionality* as a theoretical framework (Bowleg 2012), in this article we explore the intersection of culture and gambling by looking into how Pacific people’s cultural identities (reflected in cultural practices and beliefs) intersect with gambling (reflected in gambling behaviours, motivations and perceptions) to produce unique experiences of gambling harm.

The term *intersectionality* originated as a framework to explain how multiple dimensions of women’s identities (e.g. gender, age, race, ethnicity, nationality, socioeconomic status) operate interdependently (rather than in exclusive or unidimensional ways) to influence their experiences (e.g. discrimination, sexism, inequality, racism) (Bowleg 2012; Collins 2015). *Intersectionality* has since evolved from a feminist identify-focused approach to a progressing framework within other disciplines where it is applied to other marginalised groups and its scope broadened to include issues, experiences, power dynamics, and political systems (Bowleg 2012; Carbado et al. 2013; Roberts and Jesudason 2013). The framework thus offered us a lens for examining Pacific people’s cultural contexts in relation to their experiences of gambling harm. Employing intersectionality as a theoretical framework, in our article, culture is viewed as an intersectional factor in the manifestation of gambling harm, and not presumed (or proposed) to be a risk or causal factor to gambling harms. This distinction of culture is important, as cultural practices are often protective factors that contribute to community health and wellbeing (Bathgate and Pulotu-Endemann 1997; McIvor et al. 2009) and is a key feature of community social sustainability (Dempsey et al. 2011; McKenzie 2004). Furthermore, intersectionality is also advantageous in terms of framework building for future work. Clarke and McCall (2013) argued that intersectionality in social science research offers a way to conceptualise problems, formulate social explanations and offer more encompassing solutions to intersecting inequalities.

Our study adds to the limited knowledgebase about culture-related gambling harms experienced by Pacific people. We relate our findings to those described in other studies to provide a deeper level of understanding of the experienced harms by exploring how the intersection of gambling with cultural practices and beliefs may be placing Pacific people at higher risk of harm. We offer suggestions for culturally appropriate preventative interventions and harm minimisation strategies for this population.

## Methods

We analysed Pacific subset data collected in a broad study on gambling harms in New Zealand. Our purpose for Pacific-specific data analysis was to develop a deeper understanding of the cultural contexts in which gambling harm occurs among Pacific people and to expand this understanding by cross-validating findings through comparisons with those noted in previous studies. We extracted a subset of four focus groups (digitally recorded and transcribed verbatim) from the broad study to enable a focused analysis of data for culture-gambling themes specifically related to Pacific people. The focus groups were semi-structured with broad questions focusing on:

- consequences of gambling on the gambler, others close to them, and the broader community;
- negative gambling consequences that emerge at the early stage, change over time, and continue even after gambling ceases; and
- how culture constructs and influences the identified harms.

The focus group subset was selected based on the presence of participants who provided examples from a Pacific cultural perspective. Three focus groups totalling 26 professionals (i.e. budget advisors, academics, consumer representatives,^2^ clinicians and public health workers), held in December 2014, included Pacific people and others knowledgeable about Pacific people’s gambling behaviours. A fourth focus group (held in March 2015) consisted of 8 staff of Pacific problem gambling treatment services. All participants were recruited via our professional networks.

Pacific people seeking treatment for gambling problems were not purposively recruited. Nonetheless, many of the professional participants were of Pacific ethnicity and provided rich insight based on in-depth experience and descriptions of real life cases they had encountered. In particular, Pacific gambling treatment service professionals were key informants (Marshall 1996; Tremblay 1957); as reflective practitioners, of similar ethnicity to their clients, they were acutely aware of the patterns of harm experienced by their clients and were able to relate these to relevant Pacific cultural contexts. Additionally, participants representative of the range of different Pacific ethnicities were not purposively recruited, although individuals from a number of Pacific ethnicities did participate. Considering the general acceptance of Pacific people in New Zealand as having some similarities in values and cultural traits, and shared historical experiences of colonialism (Teaiwa and Mallon 2005) some generalisations are made by conceptualising traditional Pacific cultures as a whole. However, it is important to be mindful of the ethnic diversity of Pacific people and the uniqueness of each in terms of language and culture (Gray 2001; Pilato et al. 1998).

Following thematic analysis procedures, we read the transcripts several times to become familiar with the data and generate initial codes (Braun and Clarke 2006). Employing the intersectionality framework (Bowleg 2012), in our analysis, general codes on gambling (e.g. “gambling motivation”) were associated with specific cultural codes (e.g. “gift-giving”) to generate intersecting themes. Themes were identified at a latent rather than at a semantic level, as an interpretive process was used (Boyatzis 1998) to identify underlying contexts. In our analysis, a theme’s relevance was not based on its frequency within the data set, but rather on its importance (Braun and Clarke 2006) for highlighting a culture-gambling intersection. This method enabled identification of a broad range of culture-gambling intersecting themes related to gambling harms. To ensure credibility and auditability, themes are substantiated with excerpts from the raw data (Greenhalgh and Taylor 1997; Long and Godfrey 2004). To ensure authenticity, alterations to enhance sentence readability were kept minimal and participants’ exact words are provided without corrections to grammar.

## Results and discussion

**Fig. 1:**
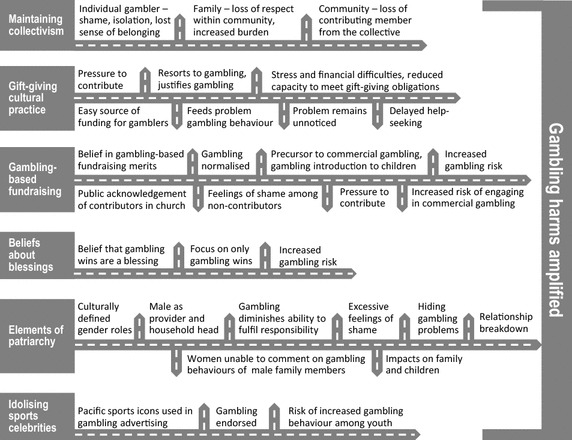
Intersections between Pacific culture (beliefs and practices) and gambling

Overall findings were indicative that Pacific people experienced gambling-related harms such as negative consequences on finances, health and relationships that are common to other populations. However, the contexts in which some harms occurred were complex and multifaceted when they were culture-related. When gambling intersected with Pacific cultural beliefs, practices and norms, the experience of gambling harm amplified as the harms branched out in different directions or manifested in multiple layers. Findings (summarised in Fig. 1) are categorised into six culture-gambling intersecting themes: maintaining collectivism, gift-giving cultural practice, gambling-based fundraising, beliefs about blessings, elements of patriarchy, and idolising sports celebrities.

### Maintaining collectivism

Pacific cultures fall within a collectivist worldview whereby Pacific people identify as being part of a large interdependent group comprising the nuclear family, extended family and lineage members from their village community (Bathgate and Pulotu-Endemann 1997; Medical Council of New Zealand 2010; Podsiadlowski and Fox 2011). In a study of collectivism in New Zealand, Pacific people demonstrated the highest preference for collectivist values and tended to distinguish the least between family, friends and strangers (Podsiadlowski and Fox 2011). Maintaining a collective society offers Pacific people a wide culture-based social support structure (Bathgate and Pulotu-Endemann 1997). Community and kinship obligations are fulfilled in several ways; for instance, helping with gardening, offering food gifts, and pooling resources to support others during weddings and funerals, in turn, strengthening interdependence between community members (Bathgate and Pulotu-Endemann 1997). Participants in our study similarly referred to the importance of collectivism among Pacific people:

… many Pacific peoples live as individuals within a collective system. So the relation or arrangements that exist between one and one’s family, one’s connection to – and when I say family, it’s not just immediate, it’s extended and it can go on and on you know. …I find [it] hard …to distinguish and separate the individual from family and collective in the Pacific context. Because they’re all so interrelated.

When we considered how Pacific collectivism (a culture-related theme) intersected with gambling-related themes, our findings showed how gambling-related harm permeated from the individual to the family and community. Gambling harm to families (e.g. financial hardships and relationship disruptions) can occur in any society regardless of culture; however, for Pacific people, collectivism meant that this harm type is more far-reaching. Harms resulting from problem gambling behaviours took on additional dimensions such as a gambler’s feelings of loss of belonging or isolation (from the collective) and shame; a family’s feelings of loss of respect; disruption of trusting relationships; transference of communal responsibilities from a gambler to family members; and an overall loss of social cohesion.

…We experienced the standard harms, as everybody else, finances, health, employment as well as employment opportunities et cetera. But … for a Pacific person, their role and responsibilities within that family context [is]…where one’s sense of belonging comes. So for a problem gambler, one of the largest harms … is when it impacts … their families and communities. …In terms of harms for the individual there’s the risk of isolating one’s self from that unit and that connectedness, that defines who that person is. So the isolation from that collective wellbeing, so to speak, is extensive. Isolating oneself from that collective also means that at times this person can’t contribute to the obligations that are associated with being part of that collective. So those pressures to contribute fall on other family members. …Because this person no longer can [contribute], there’s also a loss of respect or a sense of a loss of respect within that family group and their community. That loss of respect can have huge detrimental consequences, potential suicidal ideation et cetera. That loss of respect reflects not only on that individual, but on their family who all have the same name.

Decline in a person or family’s contribution to the community and the community’s cultural practices has been identified as a cultural harm that can result from gambling (Langham et al. 2016). Participants in our study noted how the wider community (the source of the collective identity) also experienced the loss of a contributing member.

So your attention’s diverted onto…gambling obviously. [For] other parts of the community is that effort or attention taken away from, but not in a monetary way.

…[Helping] at the old people’s home….So it’s a loss of good - opposite of harm, good stuff that they [could be] doing.

Similarly, in an earlier study, a community’s loss of contributing members (because of money lost to gambling) was identified as a reason why gambling was frowned upon among a Samoan community (Perese and Faleafa 2000). As identified in the following quotation from our study, non-present or non-contributing members may eventually end up being excluded by the wider collective.

…So, there’s that self-isolation and then it gets to a point where I guess the collective might just …not even invite anymore, because they know this person may not come along.

Weakened support networks and lack of involvement in activities of own culture have been identified as risk factors for Pacific people’s mental health (Bathgate and Pulotu-Endemann 1997). Therefore, when gambling causes an individual to disengage from cultural activities and lose support networks, it can result in a reinforcing loop that enhances the degree of harm experienced or contribute to the development of mental health issues such as depression.

The sense of responsibility in Pacific collectivism included protecting the gambler and affected family members and this sometimes meant hiding an individual’s harmful gambling behaviour. However, this may inadvertently contribute to the severity of gambling harms remaining hidden among Pacific communities.

…I know in most cases many Samoan families, they do struggle with the children being taken away from them. They don’t inform the extended family because the extended family will say, the reason why – it kind of brings shame…on the family. The reason why the kids are like that is because of the parents [gambling]. So that’s kept like a secret. So only a very few close family members will know and will do whatever it takes to try and bring the children into their care, through a family group conference, to avoid the children being [taken] into care protection and stuff like that.

### Gift-giving cultural practice

Within Pacific communities, gift-giving (e.g. *fa’alavelave* among Samoans and *fetokoni’aki* among Tongans) is among cultural practices that define the Pacific way of living (e.g. *fa’aSamoa* for Samoans and *angafakatonga* for Tongans), which is different from the European way (*angafakapalangi*) (Evans 2001; Thornton et al. 2010). Traditional gift-giving (which includes contributions to the church and at major events such as weddings or funerals) is a significant cultural practice that emphasises generosity and reciprocity, and is a means of reinforcing family relationships and maintaining social ties with extended family members, fellow church members, and friends (Evans 2001; Mulitalo-Lautā 2000; Thornton et al. 2010). Pacific people living abroad and a majority of Pacific people in New Zealand retain a strong sense of family and cultural obligations, and continue with gift-giving practices and remittances to maintain their links with family and village members in the Pacific Islands (Bathgate and Pulotu-Endemann 1997; Mcpherson 1994).

For Pacific people, meeting gift-giving obligations is regarded as important for sustaining culture-based social support structures, expressing cultural identity, receiving social recognition and maintaining self-esteem; all considered to be important protective factors for Pacific peoples’ mental wellbeing (Bathgate and Pulotu-Endemann 1997). However, as noted in earlier studies, among Pacific people, traditional gift-giving is an overt expectation that can inadvertently pressure individuals who lack the financial resources to give. These people may resort to using money allocated for essentials (e.g. education or medical treatment) or borrowing from friends, family, or fringe lenders—creating financial liability and vulnerability (Anae et al. 2007; Macpherson and Macpherson 2011). Low-income families face a dilemma when they have to choose between meeting their customary obligations or their immediate family financial needs (Bathgate and Pulotu-Endemann 1997). In a study of Pacific mothers in New Zealand (N = 1376), 62% reported gift-giving and among those mothers 59% indicated that gift-giving commitments added to their household financial difficulties (Cowley et al. 2004). Many Pacific people aimed to fulfil gift-giving obligations ahead of their household financial obligations such as paying utility bills and purchasing food (Perese and Faleafa 2000). Unlike falling back on gift-giving, there was no shame felt in not being able to pay for household expenses, although it contributed to financial hardships and affected relationships in the household (Perese and Faleafa 2000).

In our study, this culture-gambling intersection highlighted the level of pressure (including fear and shame) that the gift-giving culture can impose on individuals, inadvertently leading some to resort to gambling in an erroneous attempt to win the required funds:

… they are all pressured to give that because it’s all unshared expectations. …even if they don’t have anything they would sell whatever they have, or gamble in order to have that to maintain the relationship. Sometimes when they cannot maintain the relationship they seem to lose their credibility within their community. So it makes them look like a lesser person …They look down on themselves and…… behind the idea it’s about shame and fear…to be labelled by others, … “Oh [name] didn’t contribute much.” That kind of fear and shame will trick our people to find other avenues. …They’ll end up in - let’s say I go to a loan shark and get some money that I can’t be able to pay back, well, what else I can? …what are the alternatives? Or they end up in the casino, they end up gambling … yeah.

A lot of it’s because they still hold onto traditional ways of doing things. …It has put many, many Samoan families in poverty and struggling financially…obligations to fulfil needs to the villagers and to extended family they have never met or grown up with. Especially for weddings and funerals and hence why a lot of them, you find them at the casino and things like that.

Earlier studies have noted similar findings. In an exploratory study, Urale et al. (2015) found that financial contributions expected by distant family members motivated some Pacific people in New Zealand to gamble in an attempt to obtain extra income. In a small-sample study on motives for starting gambling, Clarke et al. (2007) found that needing money for family and to fulfil wider community obligations were significantly stronger motives among Pacific participants when compared to Māori participants. Likewise, in a study on gambling impacts on Samoan people, traditional gift-giving (often an urgent request) drove some individuals to engage in gambling activities in the hope of being able to quickly obtain the required extra cash (Perese and Faleafa 2000). This culture-gambling intersection can thus cause a negative reinforcing loop—pressure to fulfil gift-giving obligations increases risk of engaging in commercial gambling (Clarke et al. 2007; Perese and Faleafa 2000; Urale et al. 2015), which in turn increases financial risk and vulnerability, reducing (rather than enhancing) the capacity to fulfil this cultural obligation.

Our participants also reported that gift-giving obligations may be used to rationalise gambling behaviour, in turn, straining family relationships—highlighting another gambling-culture intersection.

… you know, when the mother argue with the father and she says oh you’ve been gambling a lot, the father will turn around and say hey, I’m trying to fulfil my obligation to serve. You know – hey, what else can the mother do? At the end of the day the father is working not only to cater for his family but also - when I’m talking about families, about extended families you know.

Additionally, participants reported that gift-giving practices can enable gamblers to obtain unquestioned financial support from family and friends, inadvertently allowing harmful behaviours to continue or remain unnoticed—evidencing yet another gambling-culture intersection.

In a Pacific context, our understanding of family as I mentioned earlier is extensive. …So there’s your immediate… related blood family, then there’s your village family and then your country family, on migration to a whole new country. So in essence that means a Pacific problem gambler has several people to go to. So what I’ve suggested is that delays the identification of problem gambling… Although in the Pacific context, people come and ask for money often, and it’s called fa’alavelave. Where we contribute to the collective wellbeing and to collective events et cetera. Often it’s not questioned when people come along and ask for assistance or for some monetary support. So for a problem gambler not being asked why you want money, has again huge implications for that individual, their family.

### Gambling-based fundraising

When participating in gambling-based fundraising (e.g. housie or bingo) to raise money for family, community or church, many Pacific people do not consider it to be ‘gambling’ in the conventional sense; in other words, it is thought of as different from ‘commercial’ gambling such as at a casino or pub (Guttenbeil-Po’uhila et al. 2004; Perese 2009; Perese and Faleafa 2000; Perese et al. 2009; Urale et al. 2015). Instead, gambling-based fundraising is regarded as a form of ‘giving’ or a way of ‘fulfilling’ social obligations, as the motive behind such practice is to make a donation rather than win money. An earlier exploratory study reported a belief among some Pacific people that such gambling (i.e. raffles in a church or community centre) was without losses as proceeds go into supporting an initiative of collective value (Urale et al. 2015).

In our study, this culture-gambling intersection, suggested that a belief about the merits of gambling-based fundraising could normalise gambling and result in unintended harms when individuals cannot distinguish between gambling for fundraising purposes and commercial gambling.

So when you’re talking about our Pacific communities, our churches…our Pacific gambling is not really seen as gambling. Because for years we’ve seen it, we’ve practised it as fundraising, so it’s become normalised in that way that’s how we’ve raised money for our needs, for our functions and things like that. … so if you think about where the gambling comes from or the misunderstanding of what gambling versus fundraising is…it looks different, it’s called fundraising…

Similarly, in an earlier study Pacific participants believed that the church’s acceptance of gambling for fundraising encourages or normalises gambling participation among its members and eventually leads to problematic gambling (Tse et al. 2012).

The normalisation of gambling through gambling-based fundraising also creates additional risks because it introduces and normalises gambling among children.

Many of our people go to housie [bingo], they take their children along, it happens in the islands as well. …Children sit there on the concrete floor, listening out to the housie numbers, they get a packet of chips, they get a soft drink, they’re happy. Mum and dad are playing because it’s for the good of the community. So that exists here in New Zealand as well, and it is considered acceptable because of the relationship between the minister of the church and God.

What happens in our Pacific churches with housie, is that whole families will go along. …it’s a safe environment where an older Samoan woman for example, can socialise with other older Samoan woman. Then they’ll all get together and take off to the casino for a day out. Then you take it back a step, [and look at] who’s taking our Samoan woman to the church… It’s usually the teenage adult children that will do the drop offs and the pickups, then the drop offs and the pickups to the casino. So all of that starts to, I guess you know normalise aspects of gambling and can be considered a pathway to other forms of gambling.

Evidencing another dimension of harm, when the fundraising culture intersected with gambling, it sometimes resulted in financial hardships and thus functioned as a precursor to engaging in commercial gambling activities, in turn increasing risk of financial harms.

Well the thing is, with our Pacific community most of our family and friends go to the same church you know, all of them. …At the end of the day if your aunties and uncles and friends all have the same tickets, who do you turn to, to sell those tickets to? You’re going to end up having to give your benefit money or your pay, to pay those tickets off. Then that leads back to kids going without food, you going to the pokies^3^ to try and win some money to cover the next week’s shopping or whatever.

Similarly, in an earlier study on gambling among Pacific people in New Zealand, “several participants implied that weekly offerings and tithing to churches created financial demands that could motivate one to gamble, under the assumption that gambling is a viable means of obtaining money” (Urale et al. 2015, p. 8). As insufficient income to support family and meet church and social obligations has been identified as a risk factor for Pacific people’s mental health (Bathgate and Pulotu-Endemann 1997), this culture-gambling intersection has the potential to worsen an individual’s overall health.

As identified by our participants, being unable to contribute is often associated with feelings of shame among Pacific people. Thornton et al. (2010) referred to a practice in Samoan churches, *folafola*, where the pastor announces the names of donors following a collection, as something that causes public embarrassment for those making smaller contributions. From a traditional celebratory practice of publicly acknowledging those who make substantial food and material contributions, *folafola* seemed to have transformed into a more competitive activity following Christianity acculturation and adoption of a Westernised cash economy in Samoa (Thornton et al. 2010). In our study, one participant referred to the level of shame and pressure that can be brought about by this practice in New Zealand. When unable to contribute to their church, the feeling of shame is intensified:

To save face…if you don’t sell all your tote tickets or all your raffle tickets, it’s a big shame thing within the family and a big shame thing being part of a family within the church. …I only speak for certain churches. So for my church for example, when they have these fundraisers, when we’re giving out these things we can sell, tote tickets, raffles or whatever it is, at the end of the month it’s read out in front of the whole entire congregation who has sold how many tickets and how much money they’ve brought into this fundraising for this youth trip or whatever. You can see how people feel obliged to sell everything or give all their money so that they’re not the family that hasn’t - that’s brought in the least amount of money or that hasn’t sold any tickets because it’s a big shame thing about that.

Such high levels of pressure to contribute, as identified above, can increase the risk of resorting to commercial gambling as a way for attempting to win required funds.

### Beliefs about blessings

Beliefs in the church, God and in God’s blessings are important to many Pacific people (Gershon 2007). Blessings are seen as rewards from God for good deeds or hard work that the person or family has performed. Christianity, the formal religion that most Pacific people identify with, is deeply embedded in Pacific cultures and often validates existing cultural values (Macpherson 2004). Findings in an earlier study involving Pacific people suggested how religious perceptions and practices affected gambling behaviour; for instance, a perception that commercial gambling is condoned when the underlying motive can be morally justified or when it was simply for leisure (Urale et al. 2015). Among Niuean gamblers, beliefs in blessings leads to a perception of benefits from gambling (‘wins’) as something that God predetermines and occurs when God is pleased (Collaborating Pacific contributors 2004). Similarly, among Tongan people, gambling wins are perceived as an indication of being blessed; thus, a perception that when God rewards gambling it is endorsed (Guttenbeil-Po’uhila et al. 2004). In our study, when this cultural belief intersected with gambling it gave way to potential gambling harm. One participant referred to the perception of gambling ‘wins’ as blessings as contributing harm as it influences Pacific people to only see the benefits of gambling.

So a lot of Tongan people, they look at gambling, they use the other word like moniua and they use those with in English they used the word luck, but the term moniua, it equal its meaning blessing. So if you win something, that’s how we get with their perspective. You can’t separate spirituality from them because spirituality is a big part of them. That’s how - if I win four hundred they’ll say it’s a moniua it’s a blessing from God. You see, their mindset, logically theologically it’s already there. When I, you know with my philosophical perspective I try and tell them, you know sometimes God has nothing to do with it. … it’s hard to tell them that. … with our Ministers, this is what they’ve been preaching about. You can imagine how our people are with that kind of mindset in them, so when they win the money they say oh it’s a blessing from God, but they don’t count on losing, whether it’s a blessing from God you see.

Perceptions of gambling wins as ‘gifts’ from God combined with gambling in commercial venues in order to win money needed for fulfilling obligations identified in this and other studies (e.g. Urale et al. 2015) can increase risks experienced by this population.

### Elements of patriarchy

Historically, Pacific cultures have been patriarchal or male-dominated, with clearly defined gender roles that ascribe risky and physically demanding tasks, formal political titles, and clan leadership to men, and household duties such as gardening and food preparation to women (Fischer 2013). In the island nations of the Pacific, “extended families and lineages are headed by male elders who make decisions regarding the welfare of the group” (Bathgate and Pulotu-Endemann 1997, p. 112). Today, Pacific communities retain patriarchal norms. Although increased literacy, education, employment and Western influences have caused Pacific women “to question both their Christian and pseudo-traditional obligation of subordination” and publicly demand equality, not all inherited gender roles are obsolete and men continue to direct public life throughout the entire Pacific (Fischer 2013, p. 291).

Our participants provided examples of patriarchal norms in their reference to culturally defined gender roles; males being providers and leaders within families. Perceptions of culturally defined male roles among Pacific men contributed to enhanced feelings of responsibility as head of the household and excessive feelings of shame when they were not able to fulfil their responsibility because of gambling. Therefore, when gambling intersected with the culturally defined male role this influenced the experience of harm.

The biggest saying is you’re the man. It’s the money but it’s also identity, who you are, your status, your values, integrity you know, …if I’m caught gambling, trying to put money on the – try to win money – I don’t try to win the money just for me. I’m taking my family and everything on my shoulder…so when I put money there, it’s to win the money for my family. You imagine that I lose that amount of money and I’ve ended up in a very devastating status, the amount of shame.

Violence against Pacific women perpetrated by husbands has previously been linked to the threat to male identity affected by diminished roles as income earners (Bathgate and Pulotu-Endemann 1997). Therefore, these excessive feelings of shame associated with the culturally defined male role identified in our study similarly have the potential to lead to further harms within the family unit due to possible risk of violence.

This culture-gambling intersecting theme was associated with other dimensions of harm. The enhanced feeling of shame associated with the male role was also identified as contributing to efforts to hide gambling problems (to prevent shame) and consequently to relationship breakdown.

…being a male you should be able to protect and the man starts questioning his role…it’s like putting that person against the corner so what do they do – come out fighting. To a point that everybody gets it, everybody and even himself, most times he’ll turn it back on to himself. This is to save face and this is why sometimes it stays a hidden issue for as long as they can hold it and hide it, out of the eye of the community, especially in terms of the church. It stays hidden as best as they can and everyone just lumps it until they can find help somehow along the lines and yes, that’s where the breakdown of the relationship is from.

A patriarchal culture also reduces a woman’s ability to have a say in the gambling behaviour of male family members, which could lead to prolongation and/or exacerbation of gambling harms.

…let’s say the husband or the father is the gambler, because he’s the one with the money, and the rest will, say the wife is just, her role is mainly domestic, looking after the kids, there is no voice at all. So the father makes all the decisions himself involving gambling or whatever…

He sold his son’s PlayStations and equipment like that, just to feed his addiction. He really harmed his relationship with his children, they were really angry with their father but I mean, with our Tongan context, the father is the head of the family, because they don’t have the opportunity to sit together and talk, they just keep quiet. You can imagine the silence and the amount of harm within that is huge. …they’re internalising a lot of things and it really hurts them – they can’t speak up to their father – so what they do, they go through their mum. It’s normal with a lot of our Pacific people, they go through the mother; it’s the mother who is like a mediator between the father and the children. They go through their mum and when mum is unable to speak up on their behalf so again, it ruins them internally and how they see their mum – she’s failing to stand up and they just split up.

When gambling intersects with patriarchal culture, it not only amplifies experiences of harms as identified above, but also holds potential to contribute to gambling motivation. Responses provided by female participants in Perese and Faleafa’s (2000) study as to why they gambled, suggested that they did so to gain personal autonomy and break away from traditional cultural norms imposed on them by controlling husbands.

### Idolising sports celebrities

Sports or *ta’aloga*, is an important cultural aspect within the lives of Pacific people in New Zealand and is known to provide positive experiences particularly when there is involvement from family and community (Gordon et al. 2010). In addition to improved physical health, for Pacific people, participation in sports contributes other positive outcomes including strengthened community cohesion and “development of personal and life skills such as team building, goal setting, personal discipline, self-esteem and good character” (Gordon et al. 2010, p. 4). Many of New Zealand’s rugby players are of Pacific background, whose motivation to succeed is strongly interlinked with Pacific cultural values concerning family, religion and education (Schaaf 2006). Pacific athletes are seen as cultural agents of change, who reflect what they believe to be more apt or interesting lifestyles and through whom the culture views itself (Grainger 2009).

On the one hand, idolising sports celebrities among Pacific people, may be viewed positively particularly when positive cultural values are upheld and community cohesion is strengthened. On the other hand, in a discussion on gambling marketing and advertising in New Zealand, Dyall et al. (2009) argued that sports tends to be used in subtle ways to promote gambling and engage with Pacific and Māori communities. They regarded media imaging of sports heroes (young Pacific and Māori men) supporting specific types of gambling to be risky, as sports heroes are often highly regarded as cultural icons and leaders within their communities. Endorsement of particular gambling activities by sports heroes validates the activity as an acceptable entertainment (Dyall et al. 2009). In our study, a gambling-related risk was identified in the intersection between gambling and the culturally ingrained idolisation of sports celebrities.

What comes to mind for me is the use of our celebrity sportsmen - so our Pacific men who are in the All Blacks^4^ speaking on behalf of, or you know - I suppose the casino because they’re a sponsor. But I mean, using our Pacific sports players can send a very negative message to our up and coming young sports players.

They are idolised and the community themselves will idolise them too because they’ve done well and then they can come back - can give back to their culture so yeah, and that would be a big pressure on them as well, seriously where to hide this. They have no choice, well they believe they have no choice and the commercial arm uses them that way.

The use of Pacific sports icons in gambling promotion requires further exploration, particularly with Pacific youth, since research has indicated that sports celebrities have a strong influence on youth consumption behaviours (Bush et al. 2004; Dix et al. 2010). The cultural implications of this marketing strategy are also a concern due to the potential for expansion. For instance, addressing internet gambling companies, marketing communication scholars, Prasad and Ozuem (2015) recommended investment in market research on the use of sports celebrities for online gambling advertising because of its effectiveness in engaging social gamblers.

### Cultural aspects to consider in public health messages

For effective social work practice involving Pacific people, social workers embrace Pacific conceptions of family, for instance, the Tongan concept of *matakāinga* (treating others like family), to develop mutually inclusive family-like relationships with their clients to enable positive outcomes (Mafiléo 2006). Similarly, our participants suggested that public health messages should reflect a collective identity (focusing on family and community rather than on individuals) and bridge gaps between public health workers and Pacific people. They also reported stigma associated with the term ‘problem gambling’ among Pacific people and suggested that public health messages should be framed positively, emphasising better health and wellbeing.

So the word ‘problem gambler’ is a no no to us, honestly it’s a no no. Either it’s a community colleague or a family member or a church member, or a friend. In a way we bring ourselves as the clinicians in order to go along with [the] person. So the word “we” is really important to them. When I do the public health, if I use the word ‘we’, they love to call back. If I use the word I, you, they will never call back, so that’s the difference. So the appropriate language is very important for our people, understanding them as well in order to - that’s a stigma to our Pacific people.

[We use instead]…a positive word, it’s a side-door approach, it’s a very welcoming sense of belonging including everybody for welcoming. So it never never brings the problem gambling or gambling anything. We are the example of that in order to break the cycle of stigma to the wider community…[name of our service]… means welcome, … restore health, welcome into restore health, that’s the whole vision of it.

In a previous study, participants explained how reverence towards elders in Pacific society meant that younger family members were sometimes unable to speak about the gambling behaviours of others senior to them in age even when such behaviours were causing harm (Urale et al. 2015). Our participants reported the same and suggested that advice-giving should flow from the elder to the younger.

…within the Pacific context, say mum and dad have got [gambling] problems, or an older aunty. For a younger person in that family to address that, to ask why are you asking me for money, needs to be dealt with in a particular way that’s not disrespectful, else it will just all go belly up. So just in terms of talking to somebody within that roles responsibilities and I’m here you’re here, the elders, the youngest….So it would be disrespectful first of all to be confrontational, secondly it could be considered disrespectful for you to be discussing such a sensitive topic with someone who’s not in the same age bracket as you. So yeah there would be difficulties.

This finding suggests that it may be beneficial for public health workers to be of an older age as this embraces the Pacific cultural norm of respect for elders and in turn this may increase the effectiveness potential of communicated messages.

An effective culturally appropriate model for problem gambling prevention will need to be Pacific-focused and incorporate known best practice. Knowledge could be garnered from existing Pacific health models (e.g. Agnew et al. 2004; Southwick et al. 2012), Pacific health promotion models and strategies (e.g. Bailey et al. 2010; Capstick et al. 2009; Williams et al. 2003), and cultural competence principles and communication guides recommended for health and mental health service practitioners working with Pacific people (e.g. Medical Council of New Zealand 2010; Samu and Suaalii-Sauni 2009; Suaalii-Sauni et al. 2009). For instance, differing from the conventional one-to-one healthcare model, the Pacific approach endeavours to fulfil “the multiple, layered needs of Pacific individuals, families and communities” taking into account an individual’s “family environment, community setting and cultural beliefs” (Ryan et al. 2010, p. 5). A preventative intervention could be designed along similar concepts. Public health workers may also benefit from practicing a unique indirect communication approach used by Pacific service workers that Agnew et al. (2004) refer to as the ‘roundabout’ rapport building approach, which starts with an exploratory discussion of a common interest subject with the client prior to bringing up the actual issue. This approach values Pacific relational philosophies (i.e. acknowledging a person’s status and/or hospitality prior to entering their space) and enables identification of potential barriers to working relationships with clients and/or their family (Agnew et al. 2004).

Public health workers should also consider channels through which Pacific populations are best reached. For instance, in a previous study, examples suggested for culturally appropriate awareness-raising for Samoan people included mass media and church-based programmes (Perese and Faleafa 2000). In an evaluation of problem gambling public health programs, some program implementers reported that using ethnic-specific radio channels and communicating messages in different Pacific languages was an effective way to reach Pacific communities (Kolandai-Matchett et al. 2015). However, Bathgate and Pulotu-Endemann (1997) noted that while mental health information in Pacific languages and church-based workshops are suitable for reaching Islands-born people, use of electronic media might be more suitable for reaching New Zealand-born people as less are familiar with their native languages or attend church. For these reasons, it would be important to ensure the involvement of Pacific people (both New Zealand-born and Islands-born) in the design, implementation, and evaluation of gambling harm prevention interventions. The diversity of Pacific people in New Zealand should also be considered in the design and implementation of culturally appropriate preventative interventions, as a mainstream standardised approach may not be as effective. Problem gambling preventative interventions may need be as diverse as the community needing protection.

## Conclusions

Although Pacific people in New Zealand have an overall lower gambling participation rate, population studies have highlighted a persisting higher gambling risk among this group (Abbott et al. 2014; Ministry of Health 2012). To date, very few studies have explored culture-gambling intersections that may be contributing to this higher risk. Through analysis of Pacific-specific data collected for a study of gambling harms in New Zealand, our research adds to understanding the intersection of gambling with cultural beliefs, practices and norms that can amplify gambling-related harms experienced by Pacific people. Harms were complex and multifaceted when gambling intersected with collectivism, gift-giving obligations, fundraising, patriarchy, beliefs about blessings, and idolisation of sports celebrities. Culture-gambling intersections may partly explain why Pacific people have elevated gambling risk levels and could inform the development of Pacific-specific interventions to minimise harms from gambling. Interventions should consider not just cultural concepts of gambling as an activity but also cultural concepts underpinning health, wellbeing, spirituality, connectedness and relationships to others.

## Limitations

Our findings should be considered preliminary indications of some underlying culture-related reasons for why Pacific people are at higher risk of developing gambling problems as the complexities of being a Pacific person living in New Zealand were not explored. In particular, our study did not distinguish between the different Pacific ethnicities nor between Islands-born and New Zealand born Pacific people. Instead, it was based on culturally related norms and behaviours shared among Pacific people. Further research is necessary to more fully understand the complexities of gambling harms experienced by Pacific people. The involvement of Pacific researchers would benefit these future studies in terms of data gathering, analysis and interpretation.

## Footnotes

1 The term ‘Pacific people’ is used in this paper to represent the whole of New Zealand Pacific population, which includes those born in the different Pacific Island nations and those who are New Zealand-born (Allan 2001).

2 Consumer representatives (or consumer advisors) are often individuals who have experienced gambling harms; the New Zealand Ministry of Health requires their involvement in the planning and delivery of problem gambling services (Ministry of Health 2009).

3 Pokies (poker machines) is a slang term commonly used in New Zealand and Australia in reference to electronic gaming machines.

4 New Zealand’s national rugby union team is popularly referred to as the “All Blacks” locally and internationally.
